# Quinacrine, an Antimalarial Drug with Strong Activity Inhibiting SARS-CoV-2 Viral Replication In Vitro

**DOI:** 10.3390/v13010121

**Published:** 2021-01-17

**Authors:** Mónica Salas Rojas, Raúl Silva Garcia, Estela Bini, Verónica Pérez de la Cruz, Juan Carlos León Contreras, Rogelio Hernández Pando, Fernando Bastida Gonzalez, Eduardo Davila-Gonzalez, Mario Orozco Morales, Armando Gamboa Domínguez, Julio Sotelo, Benjamín Pineda

**Affiliations:** 1 Unidad de Investigación Médica en Inmunología, Unidad Medica de Alta Especialidad, Hospital de Pediatría, Centro Médico Nacional “Siglo XXI”, Instituto Mexicano del Seguro Social, Cuauhtémoc 330, Mexico Ctiy 06720, Mexico; mony_salas@yahoo.com.mx (M.S.R.); silgarrul@yahoo.com.mx (R.S.G.); 2 Sección de Patología Experimental, Instituto Nacional de Ciencias Médicas y Nutrición Salvador Zubirán, Vasco de Quiroga 15, Mexico Ctiy 14080, Mexico; estelabini@yahoo.com.ar (E.B.); jcleonc@hotmail.com (J.C.L.C.); rhdezpando@hotmail.com (R.H.P.); 3 Laboratorio de Neurobioquímica y Conducta, Instituto Nacional de Neurología y Neurocirugía, Insurgentes sur 3877, Mexico Ctiy 14269, Mexico; veped@yahoo.com.mx; 4 Laboratorio de Biología Molecular, Laboratorio Estatal de Salud Pública del Estado de Mexico, Estado de México, Toluca 50130, Mexico; mijomeil@hotmail.com (F.B.G.); lalodvl@gmail.com (E.D.-G.); 5 Unidad Funcional de Oncología Torácica y Medicina Personalizada, Instituto Nacional de Cancerología, San Fernando 22, Mexico Ctiy 14080, Mexico; orozco81@hotmail.com; 6 Departamento de Patología, Instituto Nacional de Ciencias Médicas y Nutrición Salvador Zubirán, Mexico Ctiy 14080, Mexico; agamboad@gmail.com; 7 Laboratorio de Neuroinmunología, Instituto Nacional de Neurología y Neurocirugía, Insurgentes sur 3877, Mexico Ctiy 14269, Mexico; jsotelo@unam.mx

**Keywords:** SARS CoV-2, quinacrine, antiviral drugs, virus replication

## Abstract

Quinacrine (Qx), a molecule used as an antimalarial, has shown anticancer, antiprion, and antiviral activity. The most relevant antiviral activities of Qx are related to its ability to raise pH in acidic organelles, diminishing viral enzymatic activity for viral cell entry, and its ability to bind to viral DNA and RNA. Moreover, Qx has been used as an immunomodulator in cutaneous lupus erythematosus and various rheumatological diseases, by inhibiting phospholipase A2 modulating the Th1/Th2 response. The aim of this study was to evaluate the potential antiviral effect of Qx against denominated severe acute respiratory syndrome coronavirus 2 (SARS-CoV-2) infection in Vero E6 cells. The cytotoxicity of Qx in Vero E6 cells was determined by the MTT assay. Afterwards, Vero E6 cells were infected with SARS-CoV-2 at different multiplicities of infections (MOIs) of 0.1 and 0.01 in the presence of Qx (0–30 µM) to determinate the half maximal effective concentration (EC_50_). After 48 h, the effect of Qx against SARS-CoV-2 was assessed by viral cytotoxicity and viral copy numbers, the last were determined by digital real-time RT-PCR (ddRT-PCR). Additionally, electron and confocal microscopy of Vero E6 cells infected and treated with Qx was studied. Our data show that Qx reduces SARS-CoV-2 virus replication and virus cytotoxicity, apparently by inhibition of viral ensemble, as observed by ultrastructural images, suggesting that Qx could be a potential drug for further clinical studies against coronavirus disease 2019 (COVID-19) infection.

## 1. Introduction

We are facing a pandemic provoked by severe acute respiratory syndrome coronavirus 2 (SARS-CoV-2) as the causal agent of coronavirus disease 2019 (COVID-19) [1]. This is a β-coronavirus, with a positive-sense single-stranded RNA; it is transmitted from human-to-human through respiratory droplets [2]. Nowadays, there are no substances proven to have significant antiviral efficacy in the clinical treatment of COVID-19 infections; thus, it is a disease without specific medical therapy. SARS-CoV-2 has affinity for the angiotensin-converting enzyme 2 (ACE2) receptor, which is abundantly expressed in Vero cells (African green monkey kidney cells clone E6), which was used in this study to test the potential antiviral effect of Quinacrine (Qx) against this virus. Chloroquine (CQ) and hydroxychloroquine (HCQ) have shown modest clinical efficacy on COVID-19 patients and anti-SARS-CoV-2 effect in vitro [3,4]. Both are chemical congeners of quinacrine (Qx); however, they are considered far less active than Qx in RNA virus inhibition [5]. Quinacrine is a small molecule that diffuses freely across the membranes of acidic organelles, it is a strong DNA and RNA intercalating substance, and has been widely used as an antimalarial and anthelmintic drug, however, its clinical use has now been abandoned and replaced by other antimalarials, such as HCQ [6]. Recently, it has been proposed as a potential antiviral molecule in the treatment of human immunodeficiency virus infection (HIV), dengue virus, and Ebola virus, among others [7,8,9]. In this context, Qx has been a strong inhibitor of RNA virus replication, including Ebola virus. Additionally, Qx could modulate the immune system through phospholipase A2 inhibition, as well as TLR7 and TLR9 inhibition, and has been an effective therapy for cutaneous lupus erythematosus and other rheumatologic diseases. Currently, Qx is tested in clinical trials for prion disease and cancer. [9,10,11]. These characteristics make Qx as a potential drug to be explored against SARS-CoV-2. A recent analysis listed Qx among the top 16 repurposing drugs with potential effects against SARS-CoV [12]. In this study, we analyzed the effects of Qx as an anti-SARS CoV-2 substance in vitro, based on previous reports, pointing its apparent antiviral efficacy.

## 2. Materials and Methods

### 2.1. Cells and Virus

African green monkey kidney Vero E6 cells (American Type Culture Collection ATCC, VA, USA, no. 1586) were cultured in Dulbecco’s Modified Eagle Medium (DMEM; Gibco/Invitrogen, Carlsbad, CA, USA) supplemented with 8% fetal bovine serum (FBS; Gibco/Invitrogen, Carlsbad, CA, USA) at 37 °C with 5% CO_2_ atmosphere. A clinical strain of SARS-CoV-2 was isolated from a female patient confirmed by a real time RT-PCR test (Da An Gene Co. Ltd, Guangdong, China) and propagated in Vero E6 cells. Virus identity was corroborated by qRT-PCR with specific primers against the nucleocapsid N gene and ORF1ab gene (Da An Gene Co. Ltd, Guangdong, China). Viral infection and morphology were corroborated by transmission electron microscopy. Virus stock was maintained at −80 °C. All experiments were performed in a biosafety level-3 (BLS-3) laboratory. Virus titer was determined by quantitative real-time RT-PCR (qRT-PCR) (Takara, Mountain View, CA Cat no. 9766) with specific primers against the receptor binding domain (RBD) of viral spike gene (OriGene Technologies Inc, Rockville, MD, USA).

### 2.2. Transmission Electron Microscopy

To corroborate the morphology of the SARS-CoV-2 isolated and expanded virus, as well as to determinate the possible morphologic changes in the viral structure due to the experimental treatments, cells in a 75 cm^2^ bottle were infected with 500 μL of the viral stock at 37 °C in a 5% CO_2_ atmosphere, or with virus treated at multiplicity of infection (MOI) 0.01 with Qx 1.6 µM (CC_12.5_). The cells were collected and centrifuged 200× *g* for 15 min, after 24 h post infection. Then, embedded in Epon resin, ultrathin sections were placed on cooper grid for visualization by high-resolution transmission electron microscopy (HRTEM).

### 2.3. Evaluation of Cytotoxicity and Antiviral Activity of the Drugs

Cytotoxicity of Quinacrine dihydrochloride (Qx, Sigma-Aldrich, St. Louis, MO, USA, no. Q3251,) or chloroquine phosphate (CQ, Sigma-Aldrich, St. Louis, MO, USA, no. C6628) in Vero E6 cells was determined by the MTT reduction assay (Sigma-Aldrich, St. Louis, MO, USA). Briefly, Vero E6 cells were incubated with different concentrations of Qx or CQ (0–1000 µM). After 48 h, the medium was removed and 100 μL MTT (1 mg/mL in DMEM medium) was added to each well and incubated for 4 h at 37 °C. Then the medium was removed, and acid isopropanol was added to dissolve the blue formazan salts. Quantification of resulting blue formazan salts was done at a wavelength of 570 nm in a plate reader (EON, BioTek, Winooski, VT, USA). Results were expressed as the percentage of MTT reduction versus control values. The dose-response curves were plotted by using GraphPad Prism 6 software. The 50% cytotoxicity concentration (CC_50_) was calculated, CC_50_ value resulted from the mean value of 5 different experiments.

Vero E6 cells (1 × 10^4^ cells/well) cultured in 96-well cell-culture plates were treated with Qx or CQ (0–30 µM), for 1 h at 37 °C, together with virus at two different MOIs 0.1 or 0.01. Subsequently, the virus-drug mixture was removed; fresh medium was added and further maintained 48 h post infection. The virus yield in the infected cell supernatant was quantified by digital droplet RT-PCR (Bio-Rad, Irvine, CA, USA) and the effect of Qx or CQ in the cytotoxicity induced by SARS-CoV-2 was evaluated to calculate the half maximal effective concentration (EC_50_). The quotient of CC_50_ divided by EC_50_ gives the selectivity index (SI) value.

In order to determinate possible viral ultrastructural changes induced by viral infection and Qx treatment, a 75 cm^2^ bottle of Vero E6 cells were infected by 1 h at 37 °C together with SARS-CoV-2 at MOI 0.01 in combination with Qx (1.6 µM). Then, Vero E6 cells were collected and processed to be analyzed by HRTEM.

### 2.4. Digital Droplet RT PCR (ddRT-PCR)

Briefly, total viral RNA was isolated from cell culture supernatant using MagNA Pure LC RNA Isolation Kit-High Performance (Roche Diagnostics Co, Indianapolis, IN, USA) into the MagNA Pure LC 2.0 Instrument automated equipment (Roche Molecular System, Inc, Indianapolis, IN, USA). Quantitative RT-PCR was performed using SARS-CoV-2 N2 gene detection probe (CDC laboratory test kit for SARS-CoV-2) and One-Step RT-ddPCR Advanced Kit for Probes (Bio-Rad) on the QX ONE digital droplet PCR system. The number of viral particles were obtained using QX ONE standard software 1.0 (Bio-Rad, Inc, Irvine, CA, USA). The detection threshold and positive samples were compared by negative and positive viral control.

### 2.5. Immunofluorescence

To analyze the inhibition of viral replication induced by Qx, analysis of virions was made. Vero E6 cells (2.5 × 10^4^ cells/well) cultured in 8 well chamber slides (Nunc Lab-Tek, Carlsbad, CA, USA) were infected at MOI 0.1 and 0.01 and treated with Qx (CC_6.25_, CC_12.5_ and CC_25_). After 48 h, the cells were washed three times with pre-chilled PBS and permeabilized and fixed with fixation/permeabilization solution (BD Bioscience, Franklin Lakes, NJ, USA) for immunofluorescence analysis. Fixed cells were incubated with mouse anti-SARS-CoV-2 Spike protein S2 monoclonal antibody (dilution 1:200, Santa Cruz Biotechnology, Dallas, TX, USA) as the primary antibody, and then stained with Alexa 488-labeled goat anti-mouse IgG (1:200 dilution; Santa Cruz Biotechnology) as the secondary antibody. The cell nucleus was counter stained with DAPI. Fluorescence images were obtained by confocal microscopy (Nikon A1R HD25 microscope Melville, NY, USA).

### 2.6. Statistics

Data represent the mean ± S.D. of at least three independent experiments. The CC_50_ and EC_50_ values were calculated using GraphPad software, San Diego, CA, USA.

## 3. Results

**Figure 1:**
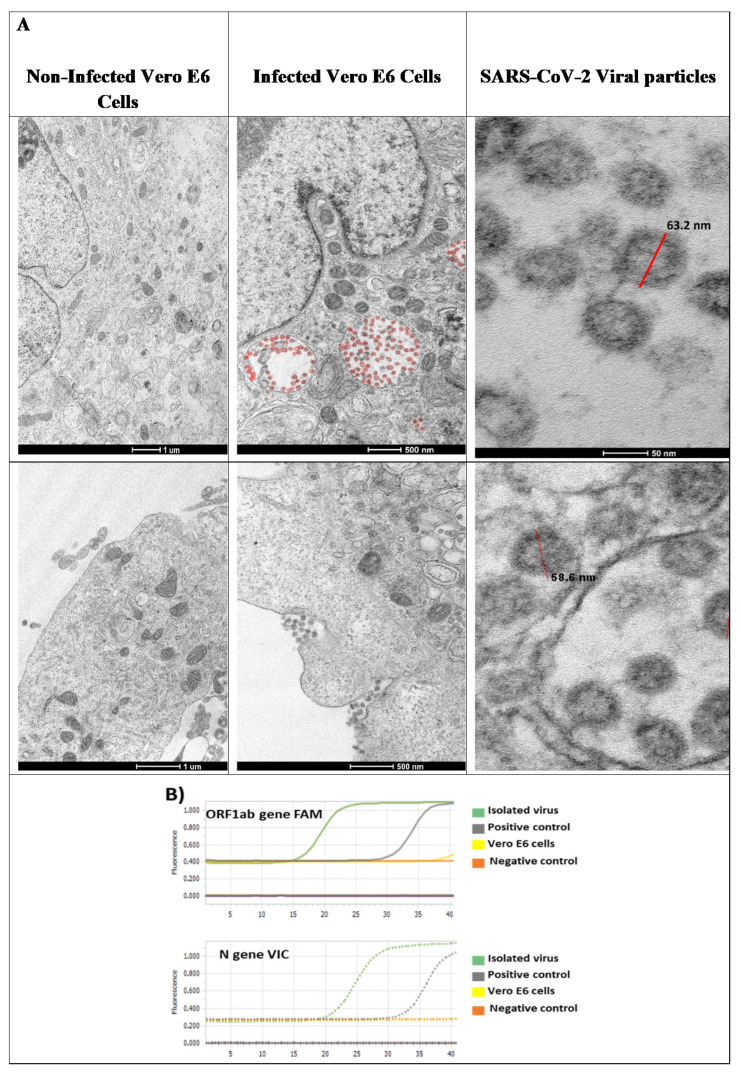
Severe acute respiratory syndrome coronavirus 2 (SARS-CoV-2) characterization in Vero E6 cells. (**A**) Viral infection of Vero E6 cells. Non-infected Vero E6 cells and Vero E6 cells, inoculated with 500 μL of viral stock, were incubated for 24 h at 37 °C, and afterwards visualized by electron microscopy. Viral particles are attached to cell membrane and endocytosed. Endocytosed viral particles (red) are internalized into early endosomes and late endosomes. Under electron microscope, viral particles with morphology similar to coronavirus 2019 are observed. Approximately 63 nm in diameter (red bar) and spike protein was observed. (**B**) Molecular identification. Isolated viruses were identified by real time RT-PCR from the viral RNA using specific probes amplifying ORF1ab gene (FAM) and N gene (VIC).

After viral isolation and cell infection, transmission electron microscopy of the infected cells was analyzed. Abundant viral particles were found over the cell membrane and contained into the endosomes (Figure 1A, second column). The viral particles were morphologically identical in size and shape to SARS-CoV-2 (see Figure 1A, last column). In contrast, non-infected control Vero E6 cells were negative for viral particles. No other types of identifiable viruses were observed.

For molecular studies, virus identification was corroborated using specific probes directed against ORF1ab and N gene, by qRT-PCR from the viral RNA; both genes were amplified, and a large number of viral particles were detected (Figure 1B).

**Figure 2:**
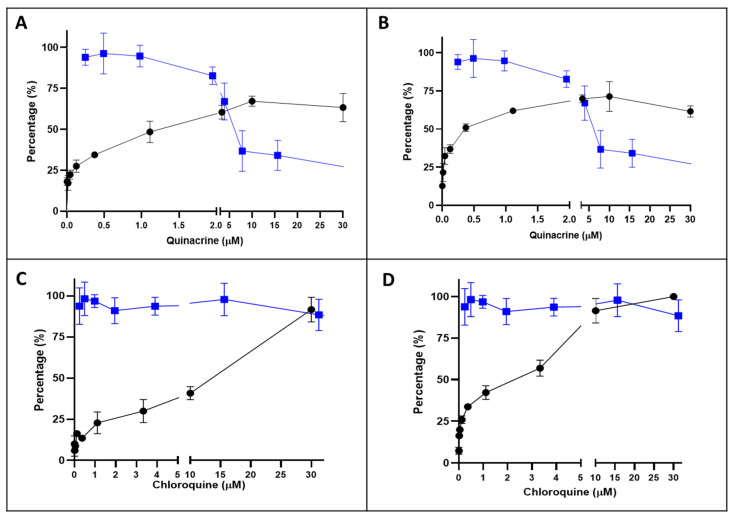
Inhibition/cytotoxicity of quinacrine (Qx) and chloroquine (CQ) in Vero E6 cells. Vero E6 cells were incubated in presence of different concentrations of Qx (**A**,**B**, blue line) or CQ (**C** and **D**, blue line) and 48 h later the cytotoxicity was evaluated by MTT. Additionally, Vero E6 cells were incubate with SARS-CoV-2 at multiplicities of infections (MOIs) of 0.1 (**A**,**C**) and 0.01 (**B**) at the same time of Qx and CQ (black lines). Data represent the mean ± SD from at least three independent experiments.

The Qx CC_50_ in Vero E6 cells, determined by MTT assay was 6.5 ± 0.8 μM (Figure 2A,B, line blue). Next, the effect Qx on the cytotoxicity induced by SARS-CoV-2 at two MOIs was evaluated (Figure 2A,B, line black). The Qx EC_50_ was 1.88 ± 0.41 µM and 0.582 ± 0.34 µM for MOI 0.1 and 0.01, respectively. The selectivity index (SI) defined as the ratio between CC_50_ and the half maximal effective concentration (EC_50_) was estimated in 3.5 for MOI 0.1 and 11.2 for MOI 0.01. As a reference control CQ was evaluated (Figure 2C,D). The CQ CC_50_ was 227.3 ± 21.7 µM (blue line) and the EC_50_ was 7.77 ± 0.852 µM and 2.85 ± 0.5171 µM for MOI 0.1 and 0.01, respectively. The CQ SI was 29.2 for MOI 0.1 and 79.7 for MOI 0.01.

**Figure 3:**
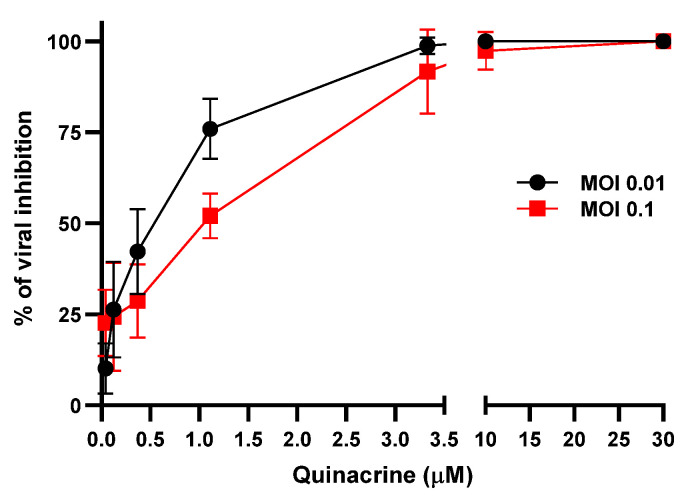
SARS-CoV-2 inhibition by Qx. Viral yield in the cell supernatant was quantified by ddRT-PCR after 48 h. Black line represent Qx effect at MOI 0.01 and red line at MOI 0.1. Mean ± SD were calculated from at least three experiments independently.

The antiviral activity of Qx against SARS-CoV-2, was measured at two different MOIs (0.1 and 0.01) and different concentrations by quantification of viral RNA copy numbers in the cell supernatant 48 h post-infection (pi.). Qx at both MOIs decreased viral copies, concentrations higher than 3.3 µM reached 99% of viral inhibition (Figure 3). The IC_50_ was 1.373 (range: 1.112 to 1.753) and 0.579 (range: 0.4106 to 0.7763) µM for MOI 0.1 and 0.01, respectively.

**Figure 4:**
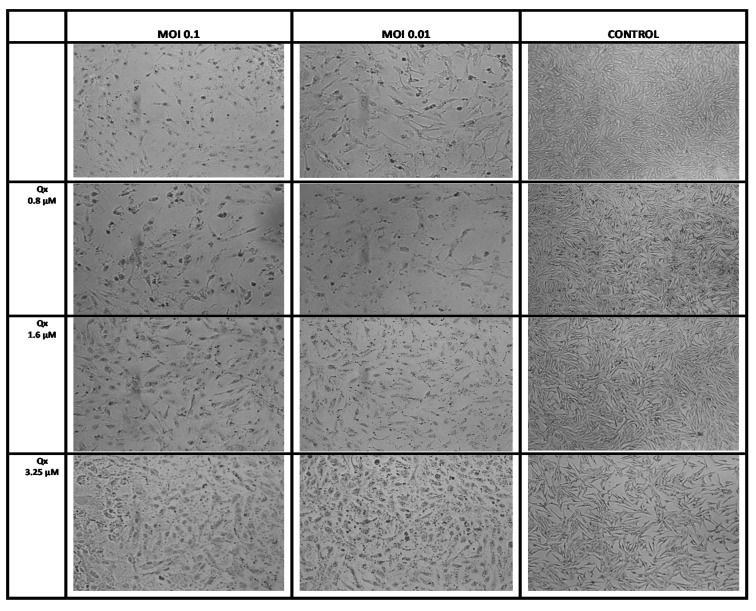
Effect of Qx against SARS-CoV-2 infection in vitro. Vero E6 cells were infected with SARS-CoV-2 at MOIs of 0.1 and 0.01 and treated with different concentrations of Qx or with PBS (controls) for 1 h, then the inoculum was changed by fresh medium and the effect was evaluated 48 h later. A representative bright field images of Vero E6 cells in each group are shown, Qx treatments protected Vero E6 cells from viral cytotoxicity. 10× magnification.

Next, we used the CC_6.25_, CC_12.5_, and CC_25_ of Qx (0.8, 1.6, and 3.25 µM) to determinate the possible Qx antiviral effect against SARS-CoV-2 infection *in vitro*. Figure 4 shows a large cell destruction and small proportion of cells attached to the well in the SARS-CoV-2 infected cell cultures (MOIs 0.1 and 0.01) without treatment. In contrast, when cells infected with SARS-CoV-2 were treated with Qx, the viral cytotoxicity decreased in a concentration dependent manner.

**Figure 5:**
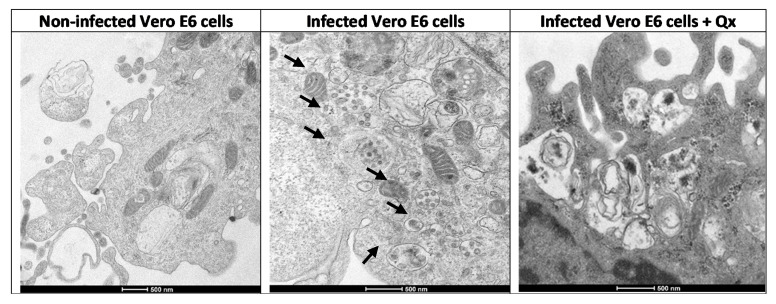
Visualization of Qx effects on SARS-CoV-2 infection by electron microscopy. Ultrathin-section electron micrographs of Vero E6 cells infected with a SARS-CoV-2 (MOI 0.01) treated with Qx (CC_12.5_), after 24 h pi. Non-infected Vero cells shows a normal cytoplasm with mitochondria, rough endoplasmic reticulum, and few vesicles. SARS-CoV-2 infected-Vero cells showed multiple assembled virions into double membrane endosomes or vacuoles, and in the vesicles (arrows). Infected cells treated with Qx shows multiple empty vesicles containing electron dense deposits, without viral particles as compared to cells without treatment (bar = 500 nm).

**Figure 6:**
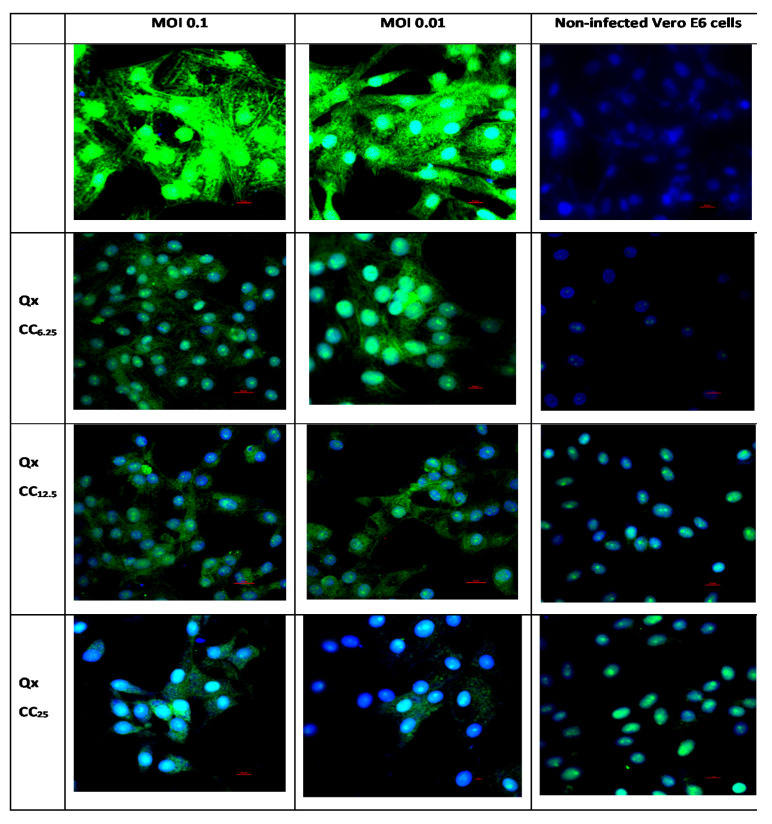
Representative immunofluorescence pictures on the effect Qx treatment (CC_6.25_, CC_12.5_ and CC_25_) in the infection of SARS-CoV-2 (MOI 0.1 and 0.01) in Vero E6 cells. Infected cells with MOI 0.1 and 0.01 are shown by the columns 1 and 2 respectively, as well as with the three different CC (6.25, 12.5, 25) for Qx treatment. Non-infected control cells are in the third column. In green: SARS-CoV-2 Spike Protein S2, and nucleus was counter stained with DAPI. Scale bar, 20 μm.

Electron micrographs of Vero E6 cells infected with a SARS-CoV-2 exhibited cytopathic effects, with multiple viral particles in the endosomes and cytoplasm; also, the complete virus particles were attached to the inner wall of the vesicles (Figure 5). The infected cells co-incubated with Qx showed multiple endosomes without viral particles, some of them associated to the endoplasmic reticulum with electron dense material, with fewer viral particles as compared to infected cells without treatment. The immunostaining confirmed a significant reduction of SARS-CoV-2 replication in Vero E6 cells treated with different concentration of Qx (Figure 6). However, cells treated with Qx showed green fluorescence in the nuclei in a concentration dependent manner, this pattern is due to the binding of Qx to DNA and RNA molecules of nuclei [13,14]. Cells treated with Qx did not show cytotoxicity.

## 4. Discussion

The COVID-19 pandemic caused by the SARS-CoV-2 infection has caused more than 1,000,000 deaths worldwide. However, even if effective vaccines are being developed, their use would not be widely available soon, and it should be considered that, even if we have them soon, these new vaccines will require low temperatures (−20 to −80 ˚C) to be preserved, which pose additional difficulties for non-developed countries. The screening of potential drugs with antiviral effects against COVID-19 is urgently needed. In this study, we evaluated the anti-SARS-CoV-2 effect of Qx, a classical antimalarial drug used for thousands of military personal for long periods, orally administered during the Second World War [15]. In our experiments, Qx showed a marked activity in vitro against SARS-CoV-2, preventing virus induced cytotoxicity and reducing viral replication. In this context, a recent study showed through network proximity analyses that Qx is indeed a potential anti coronaviruses (HCoV) repurposable drug [12]. Additionally, a virtual screening based on molecular docking has shown that Qx led to the formation of three interaction composites with the SARS-CoV-2 main protease protein [16], suggesting that this antimalarial drug could be an effective inhibitor of SARS-CoV-2.

Herein, Qx showed more than 90% viral inhibition at concentrations higher to 3.3 µM, with an estimated EC_50_ of 1.8 ± 0.41 and 0.582 ± 0.34 µM at MOI of 0.1 and 0.01, respectively. The selective index found for MOI 0.1 and 0.01 was 3.5 and 11.2, respectively, similar to those found for other FDA-approved drugs against SARS-CoV that also have shown anti-SARS-CoV-2 activity [17], but less than the SI found for CQ (29.2 and 79.7 for MOI 0.1 and 0.01, respectively). The Qx concentrations used here are clinically relevant; it has been reported that after oral administration of Qx, the drug is reasonably atoxic; it is readily absorbed in the intestine, distributed and accumulated in various tissues, and after 24 h, only 5% is excreted by urine, which is associated with a long pharmacologic half-life. The Qx concentration in plasma is very low (range 19–124 µg/L, equivalent to 0.0475–0.31 µM after oral administration of 300 mg in human) because Qx has low binding affinity to plasma constituents. However, its concentration in erythrocytes is two hundred times more than its plasma concentration. Moreover, a pharmacokinetic study in dogs has shown that Qx is accumulated 166, 556, 3926, and 1700 times more in muscle, lung, spleen, and liver than in plasma, and Qx levels in plasma are similar to those measured after the administration for malaria [18,19,20]. Based on these evidences, the concentration of Qx used in our experiments in vitro were below those found in plasma after the dose used for malaria. Moreover, recently, it was shown that Qx improves survival of mice infected with Ebola virus using i.p. doses of 25 mg/kg, which is equivalent to the doses used in humans against malaria (100 mg of Qx) [9]. Moreover, Qx permeates the brain in concentrations of 400 to 600 ng/g after a 37.5 mg/kg dose [21]; this dose could help patients with COVID-19, with various organ-related dysfunctions [22,23]. Furthermore, Qx, could decrease the cytokine storm produced in some COVID-19 patients as it modulates the immune response inhibiting phospholipase A2, reducing cysteinyl leukotriene levels, and interfering with Th1/Th2 response. In addition, Qx might inhibit the secretion of proinflammatory cytokines and Toll-Like Receptors 7 and 9 [24,25].

The therapy security profile of Qx has already being tested and allows its use in several diseases with rare and few adverse reactions, most of which are minor and reversible after discontinuation (dermatitis, corneal edema, and occasional anemia, 1 in 20,000) [26]. Recently, Qx has been tested in different studies against some cancers (ClinicalTrials.gov identifiers: NCT01839955, NCT00417274, NCT01844076) and against prion disease (NCT00183092, NCT00104663).

Recent studies have shown that Qx has antiviral effects against Zika, dengue, Ebola, and other viruses [8,9]. The mechanisms associated to the antiviral effects of Qx have been explained by various pathways: (1) its ability to intercalate to DNA and RNA molecules; (2) as a lysosomotropic agent, which increases pH in acidic organelles, modifying the enzymatic activity necessary for viral entry/cell fusion; and (3) the inhibition of autophagy-depending viral replication [8,27]. Additionally, it was reported that Qx has anti-SARS-CoV-2 activity, presumably by its binding to the ACE2 receptor inhibiting SARS-CoV-2 entry in the lung and colonic organoids [28]. In our study, when transmission electron microscopy was performed in Vero E6 infected with SARS-CoV-2, multiple assembled virions into the endosomes and vesicles could be seen, however, when Vero E6 cells were infected and treated simultaneously with Qx, multiple empty vesicles containing electron dense material were formed into the cells. This could be indicative of inhibition of assembly or maturation of viruses induced by the Qx treatment. As Qx can be incorporated into endosomes and lysosomes, raising the pH inside these intracellular compartments [29], it could lead to inhibition of protein degradation and intracellular trafficking; thus, inducing the formation of viral aggregates into the early and late endosomes. This alteration on the entry and at post-entry of SARS-CoV-2 has also been observed for CQ, as Qx similarly to CQ blocks viral infection by increasing endosomal pH required for viral replication [30].

The advantages of testing Qx as an anti-SARS-CoV-2 potential repurposing drug would be its low cost, easiness for upscale production, safety profile (already demonstrated), its effects in modulating the immune response, and its strong antiviral effect in vitro, suggesting potential advantages for clinical evaluation as potential therapy against COVID-19.

## 5. Conclusions

The results of this in vitro study demonstrate that Qx has anti-SARS-CoV-2 activity and suggests that Qx could be tested as a potential candidate for clinical therapeutic studies against COVID-19 due to various (already known) advantages of Qx therapy.

## Data Availability

The data presented in this study are available on request from the corresponding author.
